# Stimulation triggers endogenous activity patterns in cultured cortical networks

**DOI:** 10.1038/s41598-017-08369-0

**Published:** 2017-08-22

**Authors:** Valentina Pasquale, Sergio Martinoia, Michela Chiappalone

**Affiliations:** 1 Department of Neuroscience and Brain Technologies, Istituto Italiano di Tecnologia, 16163 Genova, Italy; 2 Neuroengineering and Neurotechnologies Group, Department of Informatics, Bioengineering, Robotics, System Engineering, University of Genova, 16145 Genova, Italy; 3 Biophysics Institute, Consiglio Nazionale delle Ricerche, 16149 Genova, Italy

## Abstract

Cultures of dissociated cortical neurons represent a powerful trade-off between more realistic experimental models and abstract modeling approaches, allowing to investigate mechanisms of synchronized activity generation. These networks spontaneously alternate periods of high activity (i.e. network bursts) with periods of quiescence in a dynamic state which recalls the fluctuation of *in vivo* UP and DOWN states. Network bursts can also be elicited by external stimulation and their spatial propagation patterns tracked by means of multi-channel micro-electrode arrays. In this study, we used rat cortical cultures coupled to micro-electrode arrays to investigate the similarity between spontaneous and evoked activity patterns. We performed experiments by applying electrical stimulation to different network locations and demonstrated that the rank orders of electrodes during evoked and spontaneous events are remarkably similar independently from the stimulation source. We linked this result to the capability of stimulation to evoke firing in highly active and “leader” sites of the network, reliably and rapidly recruited within both spontaneous and evoked bursts. Our study provides the first evidence that spontaneous and evoked activity similarity is reliably observed also in dissociated cortical networks.

## Introduction

*In vivo* cortical circuits spontaneously generate slow oscillatory activity in the absence of external inputs (e.g. during sleep or anesthesia^1–3^) or during quiet wakefulness^4–7^, usually referred to as UP and DOWN states^8^. Interestingly, these oscillations, in the form of synchronized bursting events alternating with silent periods, can be also found *in vitro* in isolated cortical preparations, either acute slices^9–11^ or dissociated cultures^12–15^.

The spatio-temporal activity patterns exhibited during spontaneous activity can be tracked by means of multi-channel micro-electrode array (MEA) or calcium imaging techniques and appear to be highly reliable both *in vitro* and *in vivo* ^6, 9, 11, 16–21^. A key result, reported in a variety of *in vivo* studies, is the similarity of spontaneous and sensory-evoked activity patterns^7, 22–25^, observed also in acute slices^9^. Interestingly, similarity between spontaneous and evoked activity has also been observed at a much larger scale^26^. Altogether, these findings suggest that similarity of spontaneous and evoked patterns of activity, observed at increasing levels of structural complexity, constitutes a basic and important feature of cortical function, which deserves further investigation. In this context, it has been hypothesized that cortical connectivity plays a crucial role in constraining possible activity patterns from small- to large-scale networks^26^. Luczak and MacLean^27^ proposed the idea that recurring patterns reflect the activation of specific local microcircuits. Moreover, some studies on cortical cultures^28–33^ and computational models^34^ also suggested the hypothesis that a reduced pool of strongly interconnected and highly active neurons, consistently recruited at the beginning of active states, orchestrate the spontaneous activity of cortical networks. These findings are paralleled by other *in vitro* studies on slices^35^ and *in vivo* ^36, 37^, in which spontaneous (but also sensory-evoked) activity is dominated by a small subset of highly active neurons, which are responsible for the majority of the recorded spikes and are promptly recruited in collective network activations.

In this work, we used cultures of cortical neurons plated on MEAs to study the occurrence of recurring spatio-temporal bursting patterns in *ex vivo* cortical networks, either spontaneous or evoked by electrical stimulation, and compute their similarity. To define patterns, we focused on the rank order of electrodes in burst events and improved currently existing methods based on string-edit distance measures^17, 38^ to measure rank-order based similarity. We took into consideration the role played by *leader* sites of the monitored network in coordinating both spontaneous and stimulated patterns and we asked whether the similarity between spontaneous and stimulated patterns can be related to leaders’ activation. Our results show that stimulation triggers endogenous propagation patterns with high reliability, largely independently of the stimulation source. This phenomenon is correlated to the ability of triggering firing in highly active and first-to-fire network sites, which are reliably and rapidly recruited within both spontaneous and evoked bursts. Our study provides the first evidence that similarity between spontaneous and evoked activity patterns can be observed also in generic (unstructured) cortical cultures of dissociated neurons^31, 39^, thus strengthening the idea of the ubiquitous nature of this phenomenon.

## Results

### Major leaders are reliably and promptly recruited during NBs

**Figure 1:**
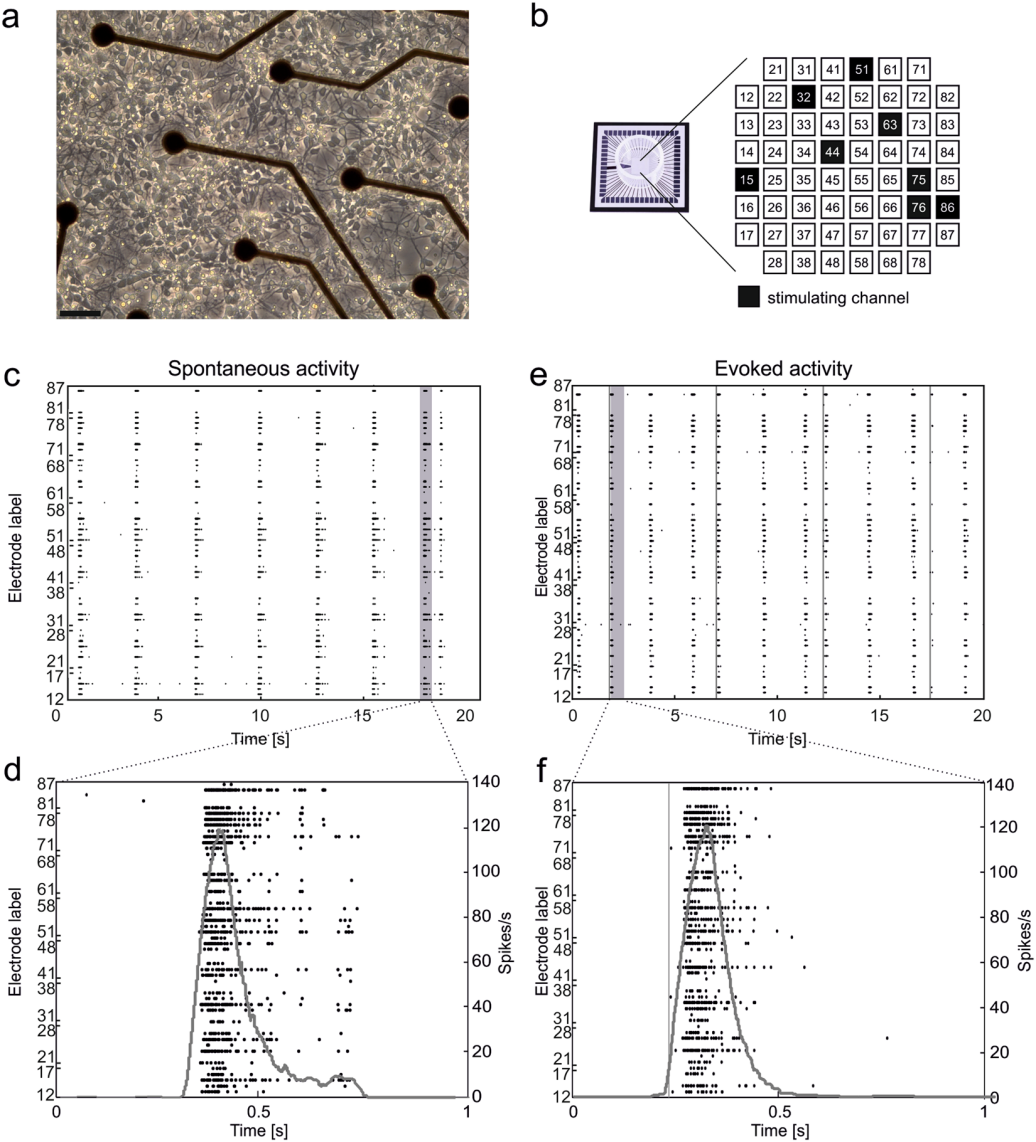
Cortical cultures over MEAs show both spontaneous and evoked synchronized network bursts. (**a**) Optical micrograph (magnification 10*x*) of a culture of dissociated rat cortical neurons plated over a MEA at 3 DIV (spatial calibration bar 50 µm). (**b**) Sketch illustrating the geometrical layout of a MEA (shown in a sample photograph on the left), including all electrodes’ labels. In each experiment, 8 different channels were selected to deliver a sequence of 100 monopolar voltage pulses at 0.2 Hz (biphasic square wave, amplitude ± 750 mV, duration 500 μs, duty cycle 50%). (**c**) Raster plot of 20 s of spiking activity recorded by a 60-electrode MEA. (**d**) Zoom on 1 s of activity highlighting a spontaneous NB. Black dots represent the detected spikes, while the grey curve is the corresponding instantaneous firing rate function (moving rectangular window: 100 ms). (**e**,**f**) Same graphical notation as in (**c**,**d**) but during a stimulation phase. Stimuli onsets are marked by grey vertical lines.

Starting from the 4^th^ week in culture, we recorded both spontaneous and evoked activity generated by cortical networks plated on planar MEAs (cf. Fig. 1a,b). After about 3 weeks of development, these networks exhibit synchronized bursting events (or network bursts, NBs) which often involve most of the recording channels of the MEAs (cf. Fig. 1c,d). Comparable events (cf. Fig. 1e,f) can be also evoked by single-pulse stimulation (cf. Methods and Fig. 1b) delivered from different electrodes.

The number of spontaneously active channels per MEA (i.e. channels having firing rate >0.1 spikes/s) was 50 ± 1 (59 available electrodes per MEA), whereas the average firing rate was 4.65 ± 0.63 spikes/s. The spontaneous rate of NBs was 19.32 ± 3.08 NB/min (min. 5.36 – max. 42.54 NB/min).

After spontaneous activity recording, we performed a preliminary detection of NBs and of major bursting activity leaders, called major leaders (MLs)^30^ (cf. Methods). Over a total of 653 active channels recorded in 13 experiments, 76 (11.64%) are classified as MLs (5.85 ± 0.53 per culture). They are usually stable over medium-term recordings (15 hours, cf. Supplementary Figure 1a) and we also know from our previous work that they tend to be conserved across development^40^. They are among the most active sites, featuring higher firing rates, higher ratio of spikes within bursts, and longer burst durations (cf. Supplementary Figure 1b,f). The presence of a few highly active and leader sites driving cortical network activity is in accordance with previous *in vitro* and *in vivo* reports^29, 32, 36, 37, 41^. Also the probability density function of logarithm of spontaneous firing rate in our cultures (cf. Supplementary Figure 1g) matched what had been previously observed *in vitro* ^42^ and *in vivo* ^43, 44^.The application of a conventional spike sorting algorithm^45^ to a subset of experiments confirms that statistically there is no tendency to classify as MLs those electrodes recording more than one single neuron (cf. Supplementary Material).

### Diverse clusters of NB patterns are associated to different MLs

**Figure 2:**
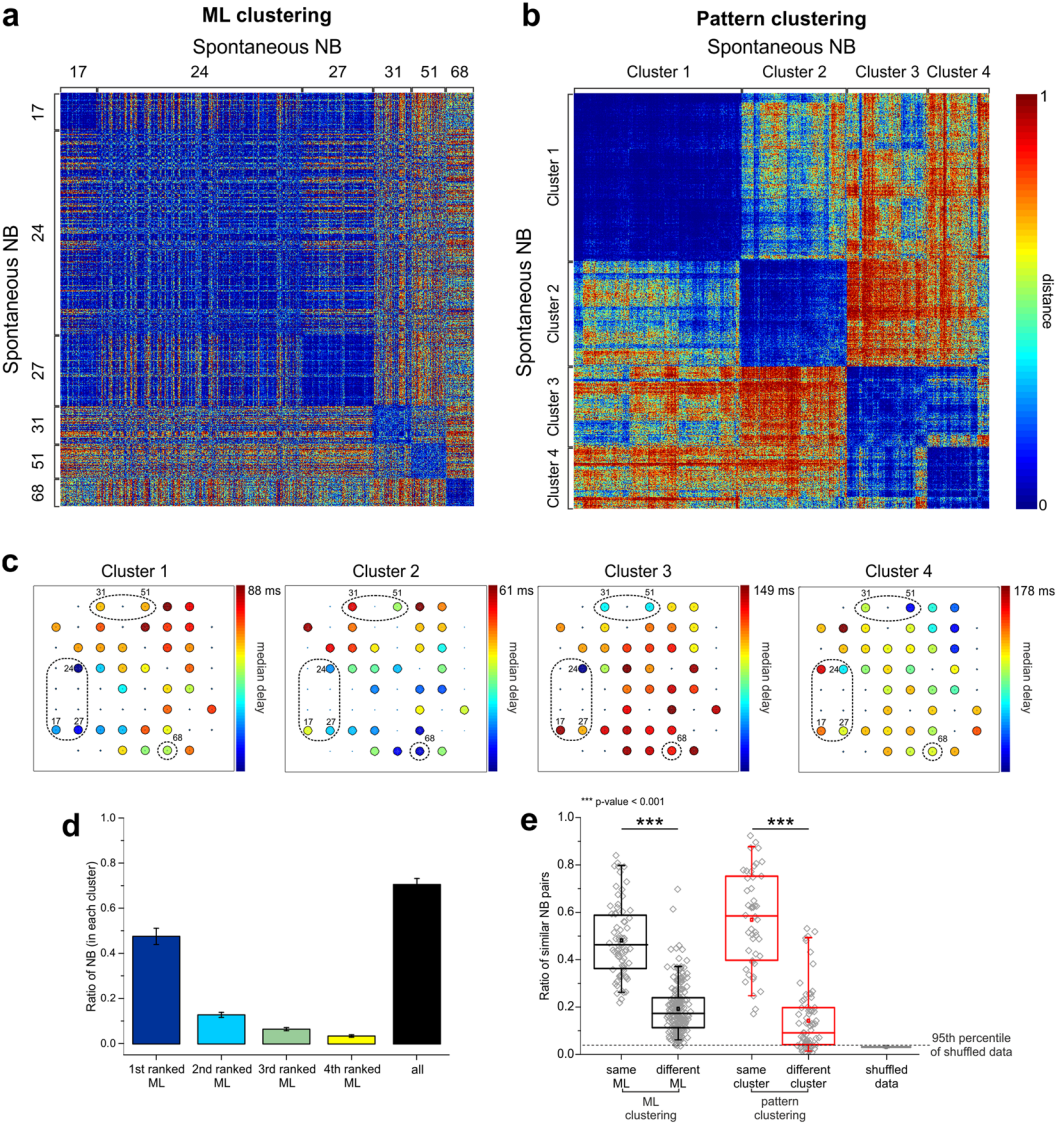
Diverse clusters of NB patterns are associated to different MLs. (**a**) Color-coded matrix of normalized distances between all pairs of spontaneous NBs (representative experiment) ordered by ML. ML numbers are reported aside, to indicate the corresponding clusters of NB. (**b**) Matrix of normalized distances between all pairs of spontaneous NBs, selected and ordered according to the pattern clustering procedure (same experiment as in **a**). In this example, four separate clusters were identified. Cluster numbers are indicated aside. Color-map: cool colors indicate low distances, whereas warm colors indicate high distances. Range: [0, 1]. (**c**) Color-coded maps of median propagation delays of electrode activations (with respect to the leader) within each cluster of patterns identified in (**b**) (same experiment). Each color-map has been rescaled according to the maximum delay, as indicated in the figure. MLs are highlighted by the dashed circles. (**d**) Bar graph of the ratio of NBs led by the four top-ranked MLs found in each cluster (colored bars). The cumulative ratio of NBs led by the four top-ranked MLs is shown in the black bar (mean ± s.e.m., full dataset). (**e**) Ratio of significantly similar NB patterns driven by the same or different MLs (black), and of similar patterns belonging to the same cluster or different clusters (red). Box plots collect data from the full dataset. The ratio of significantly similar patterns occurring by chance in case of shuffling (cf. Supplementary Methods) is reported in the grey box plot (the 95^th^ percentile of shuffled data is indicated by the dashed line). Statistically significant differences as indicated in the figure have been assessed by Mann-Whitney U-test, ${\rm{p}} > |{\rm{U}}|=0$.

We first asked whether the activation of different MLs can be predictive of the subsequent pattern of follower electrodes’ activation during NBs (i.e. propagation or activation pattern). In fact, we knew from the literature^17, 46^ that NB patterns are not completely random, but rather stereotyped, mostly belonging to just few classes of “endogenous” patterns^17, 46^. In this context, we term “endogenous” a spontaneously recurring pattern, which identifies a preferential pathway of NB activity propagation for a specific culture. In order to detect similarity among spontaneous NBs according to their pattern, we improved an existing method^38^ to compute the “edit distance” between pairs of patterns, and obtained for each experiment a “distance matrix”, i.e. a matrix collecting distances between all (pairs of) NB patterns. We first re-ordered the sequence of NBs in the distance matrix by the corresponding leader, selecting only those NBs driven by MLs (see in Fig. 2a a representative experiment, referring to ML clustering). As an alternative, we applied state-of-the-art clustering techniques^17^ to the original distance matrix in order to quantify the number of different clusters of NB propagation patterns (cf. Methods and Supplementary Figure 5), regardless of the leader. We further improved the clustering technique by adding a template-matching step, in order to cluster all similar repeating patterns and discard unclassified patterns from further analysis (cf. Supplementary Methods and Supplementary Figure 6). The result of the pattern clustering procedure, for the same experiment reported in Fig. 2a, is shown in Fig. 2b.

We can observe that, when selecting and re-ordering NBs on the basis of their ML, blue squares appear along the diagonal (i.e. collecting low distances), roughly corresponding to sets of NBs led by the same ML. Nevertheless, in some cases, NBs led by different MLs (e.g. 17, 24, 27 in Fig. 2a) seem to be remarkably similar as well. Moreover, the number of different clusters of NB patterns (cf. Fig. 2b) is usually lower than the number of identified MLs, suggesting that there is no 1:1 correspondence between MLs and activation patterns. In Fig. 2c we reported the median propagation delays of all electrodes (with respect to the first activated) in a color-coded map for the four identified clusters of patterns highlighted in Fig. 2b: cold colors correspond to shorter delays, whereas warm colors correspond to longer delays. These results suggest that different propagation patterns tend to be associated to different pools of MLs, although some MLs can also be common to different patterns. In Fig. 2d we quantified the ratio of NBs driven by the first-, second-, third- and fourth-ranked most frequent MLs within each cluster. In several cases, a high percentage of NBs belonging to the same cluster (according to pattern clustering) are led by the same ML (i.e. first-ranked ML), and most NBs in the same cluster (around 75% on average) actually start from the same pool of a few MLs (black bar in Fig. 2d). In Fig. 2e we reported the ratio of significantly similar NB pattern pairs, coming from either the same or different MLs (black box-plots), or belonging to the same or to different clusters (red box-plots, cf. Methods). NBs starting from the same ML tend to be more similar than patterns coming from different MLs, confirming that they tend to share the same following propagation pattern. The same result holds for patterns belonging to the same cluster. In both cases (i.e. bursts with the same ML, bursts belonging to the same cluster) the ratio of similar NB patterns’ pairs is markedly higher than that of shuffled data (grey box-plot).

### MLs show and evoke stronger and faster late responses

**Figure 3:**
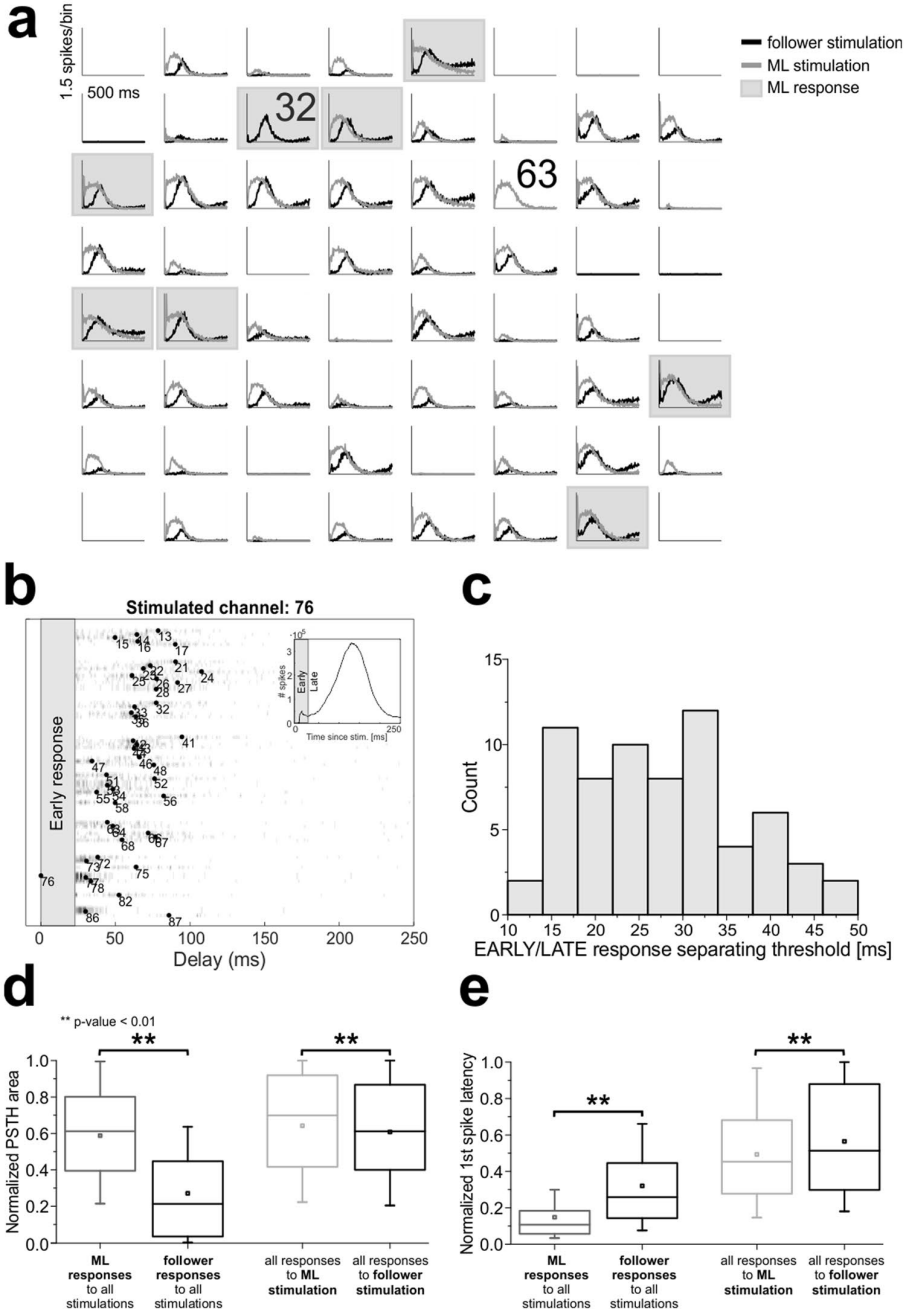
Major leaders show and evoke stronger and faster bursting responses. (**a**) Post-stimulus time histograms of all electrodes upon stimulation of channel 32 (ML, light grey traces) or of channel 63 (follower, black traces) for a representative culture. Other ML responses are highlighted by grey squares. X-scale: 500 ms, Y-scale: 1.5 spikes/bin. (**b**) Delay plot for a representative stimulating electrode: for each electrode (y-axis) we reported the histogram of delays (x-axis) with respect to the stimulus onset. Bar heights are color-coded in grey scale. Black dots indicate median delays. Electrode numbers as in Fig. 1 (**b**). The early response time period, as estimated from network PSTH (inset), is indicated by the grey-shaded area and not considered for the determination of the evoked NB pattern. (**c**) Histogram of time separation thresholds between early and late evoked response components (67 channels, all experiments). (**d**,**e**) Box plots representing statistical distributions of normalized area (**d**) and normalized first-spike latency (**e**), by considering only the late response: on the left, comparison of ML (grey) or follower (black) responses (area: Mann-Whitney U-test ${\rm{p}} > |{\rm{U}}|=0$; latency: Mann-Whitney U-test ${\rm{p}} > |{\rm{U}}|=\,1.2\cdot {10}^{-75}$); on the right, comparison of responses to either ML (light grey) or follower (black) stimulations (area: Mann-Whitney U-test ${\rm{p}} > |{\rm{U}}|=\,1.8\cdot {10}^{-5}$; latency: Mann-Whitney U-test ${\rm{p}} > |{\rm{U}}|=\,6.6\cdot {10}^{-14}$).

ML identification allowed us to deliver stimulation from either ML or follower sites (for a maximum of 8 sites, 4 of which MLs and 4 followers), to look for possible differences in the evoked activity (cf. Fig. 1b). We delivered a “test stimulus” to the culture (cf. Methods) through each of them and the evoked response was considered in a 500-ms window following each pulse. In Fig. 3a, we reported the 59+59 PSTH functions obtained by the stimulation of one ML electrode (32, grey traces) and of one follower electrode (63, black traces) in a representative culture. Other ML channels have been highlighted by a light grey square (i.e. channels 13, 15, 25, 42, 51, 78, 86). In this case, the stimulation from both electrodes is able to evoke a delayed network response involving most active channels. If we look closer to single PSTH functions, we can notice that in some electrodes the late response is preceded by an earlier and faster component, which does not always involve the same set of channels for different stimulated electrodes (i.e. early response). This early component, which had been previously associated to the direct activation of neurons by the electrical stimulation^38^, is usually comprised within a few tenths of ms after the stimulus and corresponds to a high and narrow peak in the PSTH, meaning that the activation has high reliability and temporal precision^47^.

We devised an adaptive procedure (cf. Methods), which determines, whenever possible, the optimal time threshold to separate the early from the late component for all responding channels to a single stimulated electrode (cf. Fig. 3b). We further considered those stimulated electrodes for which we could reliably separate the two components. In Fig. 3c we reported the histogram of all thresholds, showing that values are distributed between 10 and 50 ms, but are more frequent between 15 and 35 ms (entire dataset, consisting of 67 stimulating electrodes, 26.95 ± 1.15 ms; cf. also Supplementary Figure 2).

In Fig. 3d,e we reported both the normalized area (panel D) and the normalized first-spike latency (panel E) of the late response component. Same data were reported in Supplementary Figure 3a,b for the early response component. We compared either ML vs follower responses (grey vs. black box-plots) or the responses to ML vs follower stimulations (light grey vs. black box-plots). The main result is that ML responses to stimulation are stronger than those of followers, both in the late (Fig. 3d, grey vs. black box-plots) and in the early phase (Supplementary Figure 3a, grey vs. black box-plots). Moreover, responses of MLs are also significantly faster in the late phase (Fig. 3e, grey vs. black box-plots) (but not in the early one as it can be expected, cf. Supplementary Figure 3b, grey vs. black box-plots). When stimulation is delivered from MLs, they tend to evoke faster and slightly stronger late responses (Fig. 3d,e, light grey vs. black box-plots) on other channels than when followers are stimulated, although this effect is less significant than when looking at ML vs follower responses (cf. caption of Fig. 3). Finally, ML stimulation is also able to evoke faster (but not stronger) early responses (Supplementary Figure 3a,b, light grey vs. black box-plots).

In summary, we found that MLs are similarly activated within both spontaneous and evoked NBs, and their stimulation is more effective in triggering network responses.

### Evoked NBs from different stimulated channels share quite similar patterns

After characterizing the average properties (strength, latency) of ML responses to stimulation and of responses evoked by ML stimulation, we applied the same clustering procedure to evoked NB events (cf. Supplementary Figure 6), separately for each stimulated channel’s responses. We observed that stimulation from a single source mostly evokes a single reliable activation pattern, as also reported previously in the literature^38^. The goal here is to identify the main propagation patterns of NBs evoked from different sources.

**Figure 4:**
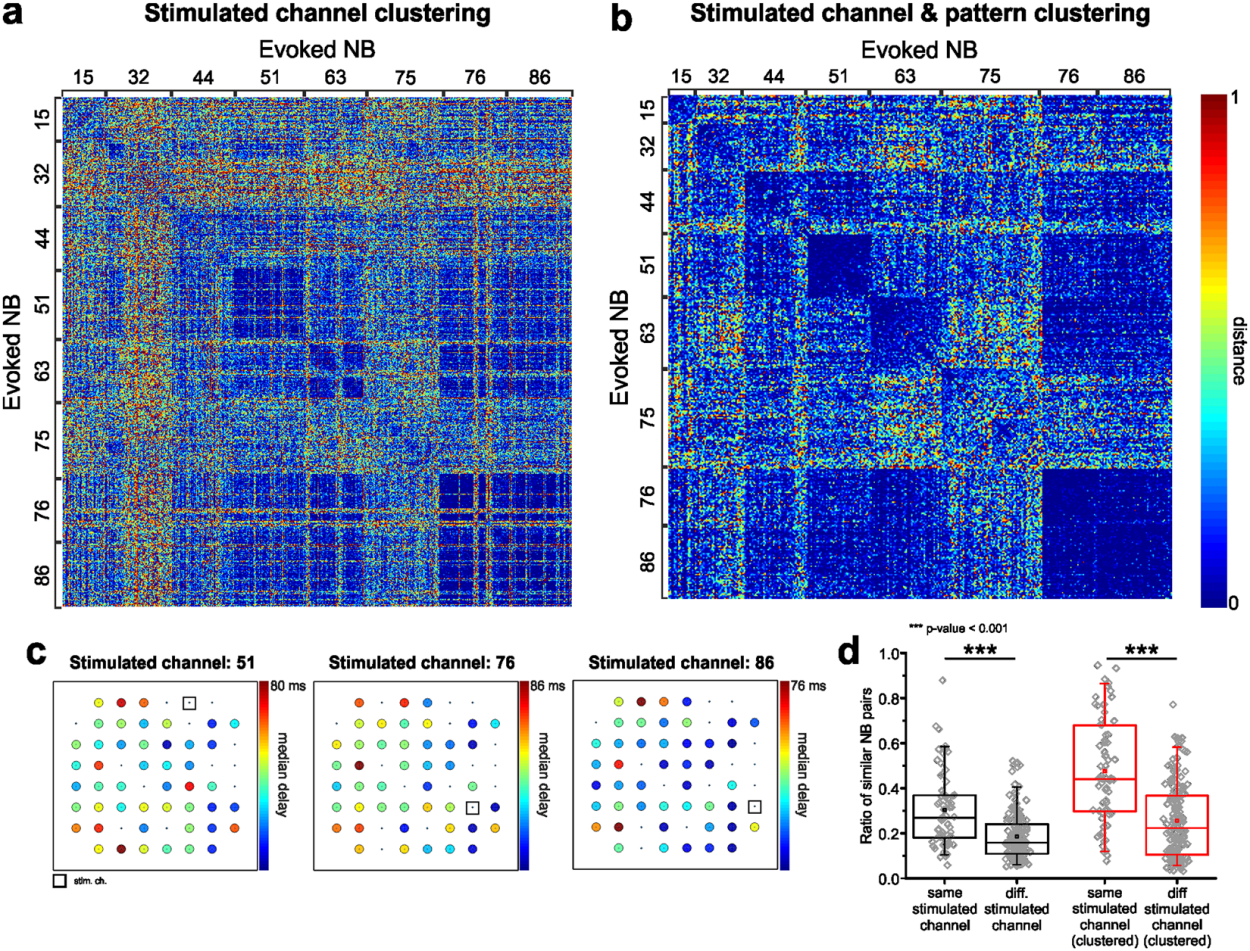
Evoked NBs from different sources share similar patterns. (**a**) Color-coded matrix of normalized distances among all pairs of evoked NBs (representative experiment) ordered by the corresponding stimulated channel. (**b**) Matrix of normalized distances among all pairs of evoked NBs (same experiment as in **a**) ordered by the corresponding stimulated channel and selected according to the pattern clustering procedure, applied separately to each stimulated channel’s responses. Stimulated channels’ numbers are reported aside, to indicate the corresponding clusters of NB patterns (also in **a**). Color-map: cool colors indicate low distances, whereas warm colors indicate high distances. Range: [0, 1]. (**c**) Color-coded maps of median propagation delays of electrode activations (with respect to the stimulus onset) for three different stimulated electrodes (same representative experiment as in **a**,**b**). Each color-map has been rescaled according to the maximum delay, as indicated in figure. (**d**) Ratio of significantly similar patterns evoked by the same or different stimulated channels, either considering all patterns (black) or only the clustered ones (red). Box plots collect data from the full dataset. Statistically significant differences as indicated in figure has been assessed by Mann-Whitney U-test (all patterns: ${\rm{p}} > |{\rm{U}}|=\,2.1\cdot {10}^{-8}$, all clustered patterns: ${\rm{p}} > |{\rm{U}}|=\,5.0\cdot {10}^{-11}$).

In Fig. 4a, we reported the distance matrix between all pairs of evoked NBs clustered according to the corresponding stimulated channel, and in Fig. 4b the one resulting after selecting patterns based on our clustering procedure (representative experiment). In this case, the clustering procedure identified the most frequent propagation pattern evoked by stimulation of a single channel and discarded unclassified patterns. In Fig. 4c we showed the color-coded propagation delay maps of the selected evoked NBs from three different sources (electrodes 51, 76 and 86, same culture as in Fig. 4a). In Fig. 4d we compared the distributions of the ratio of similar NB patterns driven by the same or different stimulated channels (13 cultures, 67 stimulated channels), and either applying (red box-plots) or not applying (black box-plots) the clustering procedure. We observed that patterns elicited by different stimulation sources seem to share a considerable degree of similarity, as qualitatively illustrated also in panels a and b of Fig. 4: low distances (i.e. cold colors) between pairs of patterns elicited by different stimulation sources indicate that they are actually similar. Also the propagation delay maps show that in this representative culture the bursting responses tend to start in the bottom right corner and to propagate leftward, even if with slightly different absolute delays of electrodes’ activations. The global population statistics (cf. Fig. 4d) shows that although there is a significant statistical difference, patterns evoked by different sources are less separated among themselves than spontaneous patterns belonging to different clusters (median ratio of similar evoked NB patterns from different stimulated channels 0.22, median ratio of similar spontaneous NB patterns belonging to different clusters 0.09, Mann-Whitney U-test $p > |U|=\,3.0\cdot {10}^{-8}$). Moreover, the application of the clustering procedure enhances not only the reliability of patterns from the same source, but also of patterns from different sources (median ratio of similar evoked NB patterns before clustering 0.16, after 0.22).

### Spontaneous and evoked NB patterns are similar

**Figure 5:**
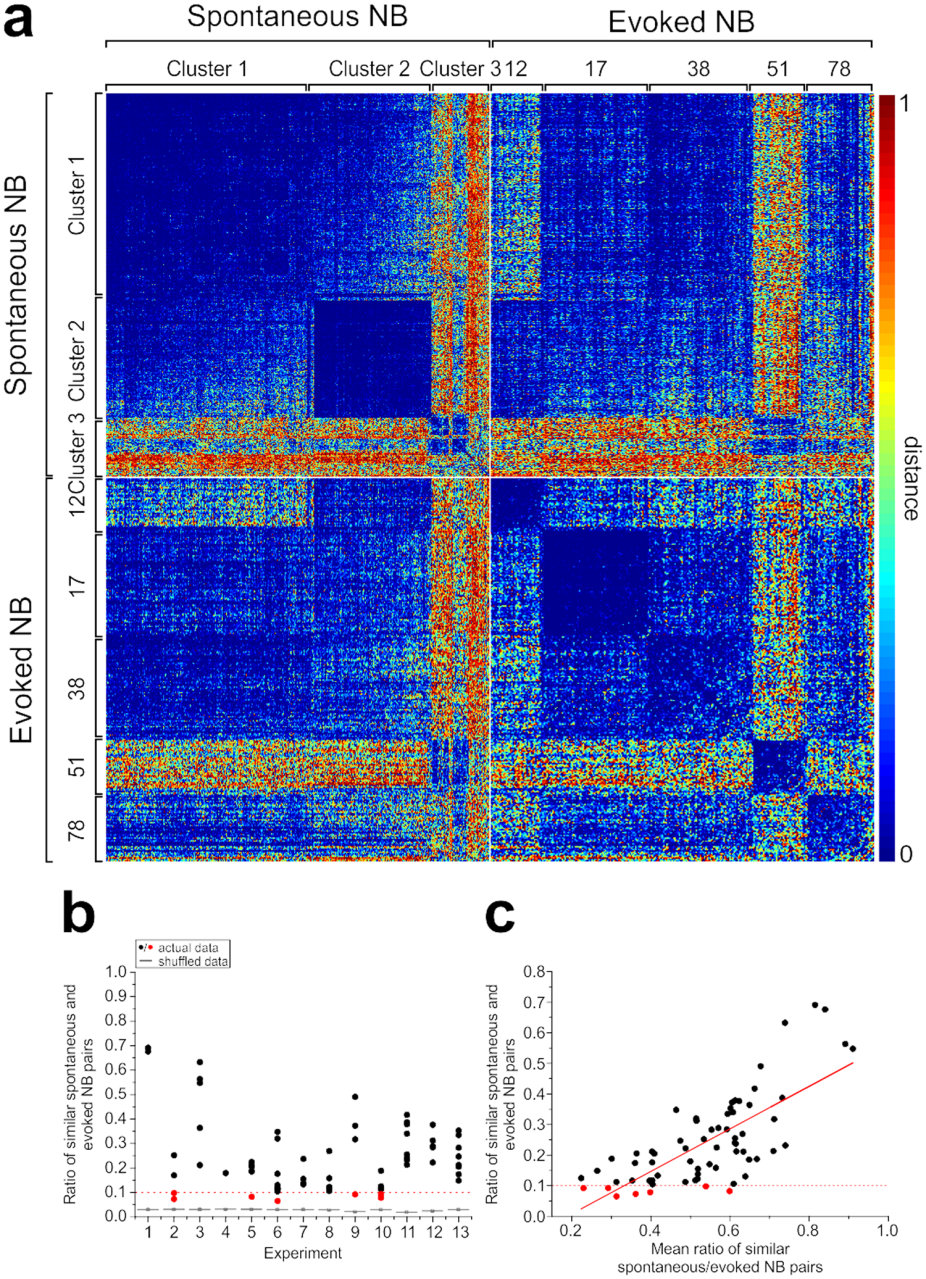
Spontaneous and evoked network bursts show similar activation patterns. (**a**) Color-coded matrix of normalized distances among all pairs of NBs, both spontaneous and evoked, for a representative experiment. NBs have been re-ordered according to the pattern clustering procedure, run separately on spontaneous and on evoked patterns. Cluster and stimulated electrode numbers have been reported aside. (**b**) Maximum ratio of similar spontaneous and evoked NB patterns (as a measure of similarity), for each stimulated electrode as a function of the experiment number (x-axis). Each point represents a stimulated electrode, in black showing ratio >0.1, in red ratio <0.1. Statistical distributions of the same parameter computed between shuffled spontaneous and evoked patterns are reported for each experiment in the grey box-plots. The grey shaded area highlights the experiment shown in (**a**). (**c**) Scatter plot of the ratio of similar spontaneous and evoked patterns (already shown in **b**) as a function of the mean ratio of similar patterns in the corresponding spontaneous and evoked clusters. The red line indicates the best linear fitting (Pearson’s correlation coefficient 0.73, ${\rm{p}}\,=\,2.85\cdot {10}^{-12}$, adjusted R-square 0.53).

In light of the previous results, we hypothesized that the similarity between evoked NB patterns from different sources could be explained by the fact that the same endogenous pattern is actually triggered independently from the stimulated channel. Therefore, we computed the distances between each pair of spontaneous and evoked NBs. In Fig. 5a we reported for a representative experiment the full matrix of distances between all pairs of patterns, considering both spontaneous and evoked NBs. Pattern clustering had been applied previously and independently to the two groups of spontaneous and evoked NB events. Hence, spontaneous and evoked NB patterns have been selected and re-ordered independently of their cross-distances (cf. Supplementary Figure 6).

We can notice that also distances between spontaneous and evoked patterns are remarkably (and also significantly) low (cf. second and third quadrant of the full distance matrix in Fig. 5a), as it happens for distances between pairs of evoked patterns from different sources. For each pair of spontaneous and evoked clusters of patterns we computed the ratio of significantly similar patterns’ pairs over the total number of pairs and we determined its maximum value for each stimulated channel (reported in Fig. 5b as a function of the experiment number). This allowed us to associate each evoked pattern to the most similar cluster of spontaneous patterns. We can regard this as a quantification of the “similarity” between spontaneous and evoked NB patterns for each stimulated channel. We also computed the same quantity when shuffling spontaneous patterns several times and reported its distribution for each experiment in the grey box plots of Fig. 5b. It is evident that the ratio of similar spontaneous and evoked patterns is much higher than expected by chance in most cases (higher than a conservative threshold equal to 10% in 60 out of 67 channels, 89.55%). In 8 out of 13 cultures for no tested channel this parameter was less than 10% (61.54%).

We also used an alternative method to visualize and quantify the similarity between spontaneous and evoked patterns, based on the multidimensional scaling analysis technique (cf. Supplementary Methods and Supplementary Results). Results in Supplementary Figure 4 confirm the similarity of spontaneous and evoked patterns, showing that the realm of spontaneous patterns subtends possible evoked patterns. Finally, we correlated the ratio of similar spontaneous and evoked NBs to the ratio of similar NB patterns within each of the two corresponding spontaneous and evoked clusters (by computing the average of the two). Those data are reported in Fig. 5c as a scatter plot. We found a significant correlation between these two quantities (Pearson’s correlation coefficient 0.73, p = 2.85·10^−12^), meaning that the more reliable spontaneous and evoked propagation patterns are, the more similar they will be among themselves.

### MLs involvement in evoked patterns

We asked whether spontaneous vs. evoked patterns’ similarity could be different when stimulating either MLs or followers, but we could not find any significance when comparing the statistical distributions of the ratios of similar spontaneous and evoked patterns in the two cases (two-sample t-test, p $> |t|=\,0.88$). This is consistent with the observation that the stimulation source does not fully determine *a priori* which pattern is generated, and that there is remarkable similarity among evoked patterns from different sources.

**Figure 6:**
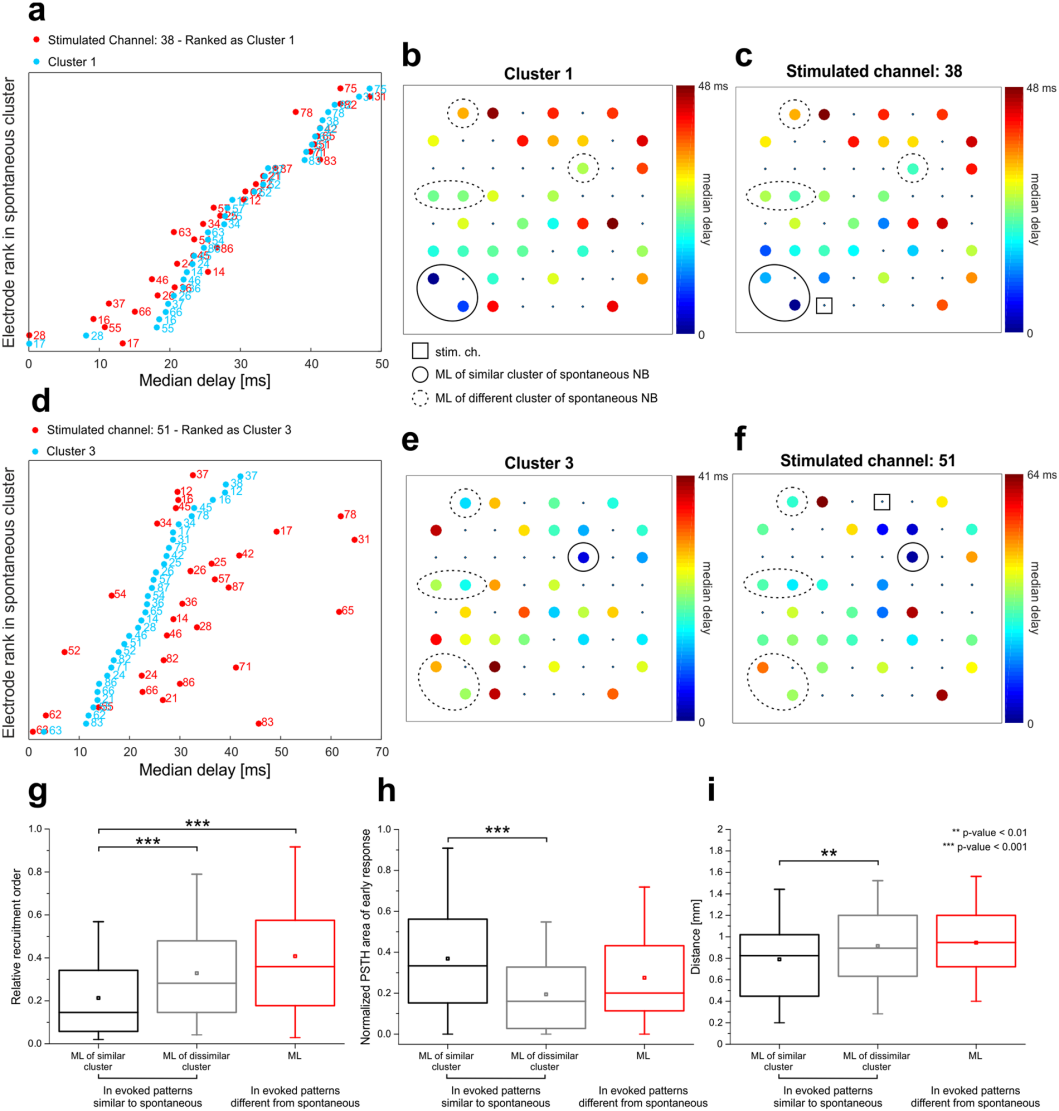
ML prompt and direct activation is predictive of pattern similarity. (**a**,**d**) Median delays of activation for two different stimulated electrodes (in red) and the associated similar spontaneous pattern (in light blue, same experiment as in Fig. 5a). For each electrode (on the y-axis) we reported the median delay of the first spike with respect to either the stimulation onset or the first activated electrode (on the x-axis). Electrodes, identified by a label according to the MEA layout (cf. Fig. 1), are sorted based on the rank order of median delays observed in the spontaneous cluster. (**b,c,e,f**) Color-coded maps of the median propagation delays of electrode activations (with respect to either the stimulation onset or the first activated electrode) for the same two stimulated electrodes and the corresponding similar spontaneous pattern. Each color-map has been rescaled according to the maximum delay (in ms), as indicated in figure. MLs and the stimulated channel are highlighted as indicated in the legend. (**g**) Statistical distributions of relative recruitment order of MLs in evoked patterns. (Kruskal-Wallis ANOVA on ranks with Dunn’s correction for pairwise multiple comparisons, p < 0.001). (**h**) Statistical distributions of normalized PSTH area of early response of MLs in evoked patterns. (Kruskal-Wallis ANOVA on ranks with Dunn’s correction for pairwise multiple comparisons, p < 0.001). (**i**) Statistical distributions of geometric distance of MLs from stimulated channels in evoked patterns (Kruskal-Wallis ANOVA on ranks with Dunn’s correction for pairwise multiple comparisons, p = 0.005).

Then, we asked whether the early involvement of MLs in evoked responses was predictive of the similarity between evoked and spontaneous patterns led by the same MLs. In Fig. 6a–c we show for a representative experiment (the same used in Fig. 5) that stimulation of channel 38 reliably evokes a pattern which is similar to the spontaneous one identified as Cluster 1 (roughly corresponding to MLs 17 and 28, the closest ones to the stimulated channel). The same happens when stimulated channel 51 in the same culture (cf. Fig. 6d–f), evoking a propagation pattern similar to spontaneous Cluster 3 (starting from ML 63, again the closest ML to 51).

These qualitative observations are quantified in the panels of Fig. 6. First of all, we divided evoked patterns in two categories, either *similar to spontaneous* (whose corresponding ratio of similar spontaneous and evoked patterns is higher than 10%, cf. Fig. 5, 60 channels out of 67) or *different from spontaneous* (ratio of similar spontaneous and evoked patterns lower than 10%, 7 channels out of 67). Then, in evoked patterns similar to spontaneous, we considered separately either the MLs driving the similar spontaneous cluster or the MLs of the dissimilar clusters. For all the other patterns, we considered MLs altogether. When looking at the relative recruitment order of MLs in evoked patterns, we found that MLs leading the associated spontaneous cluster feature significantly lower recruitment orders than all other cases (cf. Fig. 6g). This result could be expected, given the similarity between spontaneous and evoked patterns and the fact that MLs are recruited with shorter latencies to stimulation onset (cf. Fig. 3e). Then we looked at the normalized PSTH area of MLs, but considering only the early component of the response, the one which precedes the generation of the bursting response and that was not considered in the determination of the evoked activation pattern. In this case, MLs of similar endogenous patterns show higher PSTH areas than MLs of dissimilar ones (cf. Fig. 6h), meaning that when stimulation “directly” activates MLs (i.e. directly elicits a higher number of spikes on MLs) the corresponding endogenous pattern is more likely to be subsequently evoked in the late response phase. Finally, we also looked at the geometric distance between stimulated channels and MLs, and we found that when a stimulated channel is closer to a ML, there is a higher chance that the corresponding endogenous pattern is evoked. In fact, MLs of similar spontaneous clusters are significantly closer to stimulated channels than MLs of dissimilar clusters (cf. Fig. 6i).

In summary, when stimulation is able to directly evoke firing in MLs, which are by definition reliably and promptly recruited during spontaneous NBs, the corresponding subsequent endogenous pattern is more likely to be generated.

## Discussion

Our study was aimed at investigating the relationship between spontaneous and evoked synchronized network events. We first analyzed the spontaneous activity of cortical cultures and, in agreement with previous reports^30, 31^, we found that a few electrodes record earlier and sustained activity during synchronized bursting (i.e. MLs). Then, we asked what relationship there is between the occurrence of a specific activity pattern and the early activation of MLs, finding that diverse activity patterns are associated to the activation of different pools of MLs. Since MLs appear to be strongly and promptly activated also during electrically stimulated events, we extended our analysis to evoked patterns and asked what role MLs play in the generation of bursting responses. We first showed that stimulation actually triggers NBs which strongly resemble endogenous (i.e. spontaneously generated) ones, independently of the stimulation source, and then demonstrated that this phenomenon is mediated by the capability of stimulation to elicit early (direct) responses on the corresponding pool of MLs. We interpreted these results as a supporting evidence of the existence of preferential micro-circuits underlying the emergence of cortical recurring patterns, as previously hypothesized in^27^. These findings are in agreement with numerous previous experimental (both *in vitro* and *in vivo*) and computational reports (see Introduction), linking for the first time the two previously independent findings, i.e. the generation of reliable sequential patterns and the evidence of *sparse* firing in the neocortex^36, 41^.

The experimental evidence for the presence of highly active neurons in the functional organization of cortical micro-circuits is accumulating very rapidly: *in vitro* those neurons have been usually identified by their leadership property, either called “major burst leaders”^29, 30^, or “first-to-fire”^31^, or simply “highly active”^32^. *In vivo* many recent studies reported about the presence of a minority of neuronal cells which account for the majority of recorded spikes, as an evidence of *sparse* firing in the neocortex^36, 41, 43, 48^. In fact, when computing the probability density function of logarithm of spontaneous firing rate in our networks we found the same pseudo log-normal distribution derived in a theoretical model^44^ and found both *in vivo* ^43^ and *in vitro* ^42^.

Here, we focused on the presence of major leaders^30^, which also corresponded to many of the most active sites in the network, showing not only higher global firing rates, but also longer burst durations and lower percentage of desynchronized spikes. These results are in accordance with what was previously shown in other experimental studies on the same kind of preparation^29–31, 42^, and confirm that some electrodes in the array (i) are consistently activated in the first stages of bursting activity propagation and (ii) feature higher firing rates. Given the reduced number of recording electrodes of MEA devices (60 electrodes over an area of about 1.5 mm^2^) and thus estimating that less than 1–5% of neurons in the network are actually sampled, there is a high chance that MLs are simply the first activated sites recorded during bursts and that the propagating activity is starting outside the monitored area of the network. However, they belong to a sub-population of cells which are consistently recruited into collective events in the first stages of propagation, although there is no proof that they actually ignite them (see Discussion of refs 30, 32). Moreover, other studies, using calcium imaging to monitor cultured cortical networks and to overcome MEAs’ limitations in terms of spatial resolution, reported about the existence of a few discrete areas of the network that control the activity of the entire culture^28, 39^.

The firing properties of MLs are also maintained in delayed evoked burst events, since they show higher numbers of evoked spikes and shorter latencies to stimulation. They also respond better to stimulation in the early phase, suggesting that they are strongly coupled to many physical locations in the network^32^. Moreover, when stimulation is delivered from MLs, responses show shorter latencies (both early and late), indicating that the network is faster entrained by ML than by follower stimulation.

These observations raise the question of what distinguishes MLs from other units in the network: the most accredited hypothesis is that they feature different structural (and hence functional) connectivity. Effenberger and colleagues^34^ presented an adaptive model of balanced neuronal network featuring STDP and synaptic scaling, in which a highly connected subnetwork of *driver* neurons with strong synapses emerges as a consequence of self-organization following synaptic plasticity rules, as well as long-tailed distributions of synaptic weights and firing rates. Moreover, coincident activation of several driver cells is able to elicit population bursts in the model. Other theoretical studies confirm that leader neurons can be distinguished on the basis of their structural connectivity, e.g. featuring higher excitatory in-degrees^49, 50^. Inhomogeneous functional connectivity, showing high-clustering and modular small-world organization, has been found experimentally both in cortical and hippocampal cultures^51–53^. Schroeter *et al*. interestingly found in hippocampal networks a pronounced rich-club topology, where hubs tend to connect among themselves and, at the same time, act as *brokers* of spontaneous multi-unit activity^53^.

To measure pairwise similarity among activity patterns, we refined a method previously presented by Shahaf and colleagues for the analysis of evoked events^38^ in which (i) we implemented a better separation of early and late response components by means of an adaptive procedure and (ii) we introduced a normalization procedure based on surrogate data generation via random shuffling. Normalization allows to highlight similarities between patterns regardless of the length and enables the successful application of the following unsupervised clustering procedures. Differently from other approaches to the computation of pattern similarity based on the analysis of propagation delays^17^ or of the spatial center-of-mass of activity^16^, here the only variable that matters is the activation rank order of electrodes during NBs. An alternative measure of rank order pattern similarity could be the Spearman’s rank correlation coefficient^54^. However, to determine significance (and confidence intervals) of correlation coefficients, sample size would be an issue^55^ and approaches based on permutation tests to compute null hypothesis distributions might still be preferable, because they automatically take into account the sample size. Also our method implies the generation of high-number surrogate data in order to “normalize” edit distances and test their significance, thus requiring long computational times for numerous datasets. However, given the availability of modern high-performance computation facilities, this cannot be considered anymore a real bottle-neck.

When focusing on evoked NBs, the main result is that evoked patterns are strongly similar to endogenous ones. This result about similarity of spontaneous and evoked events in cortical micro-circuits is consistent with previous findings in thalamo-cortical slices *in vitro* ^9^ and in *in vivo* auditory cortex^7^. The main underlying hypothesis is that recurring activity in local cortical micro-circuits forms quasi-stable *attractor* networks whose repeated activation is reflected in cortical UP state propagation^11, 56, 57^. The same attractors would draw network activity during sensory-evoked events, regardless of whether the stimulus has been experienced before or not by the animal^7^. The fact that in our experimental model system the structural and functional organization of cortical micro-circuits is not retained from *in vivo* supports the robustness and universality of this self-organized phenomenon. Its functional significance *in vivo* has been well described in^7^, particularly in terms of redundancy and robustness of information encoding. In this view, recruitment order can still be considered a candidate neural ensemble code of sensory information^38, 58^, provided that different stimuli induce small timing variations within broadly conserved sequential patterns, which could still be detected by downstream structures for further processing^7, 59, 60^. This hypothesis seems to be confirmed by our data, since activity patterns evoked by the same stimulation source show higher similarity among themselves than with patterns elicited from different sources and endogenous ones, leaving open the possibility to be recognized as distinct patterns (e.g. by supervised learning techniques, as done by ref. 38).

Our results are also in agreement with another recent study which postulates that strong spatio-temporal localization of the noise-driven activity due to sensitive noise amplification is actually responsible of spontaneous activity generation in generic cortical micro-circuits^39^. The hypothesis is that what promotes a given region into a strong “nucleation” site is the massive confluence of paths of large amplification. The result that stimulation is more likely to activate the same preferential paths of propagation of spontaneous activity, with higher probability when leader sites are directly activated in the early response, is compatible with this view.

## Methods

### Cortical culture preparation

All experimental procedures and animal care have been approved by the IIT Animal Welfare Body and by the Italian Ministry of Health (authorization 110/2014-PR), in accordance with the National Legislation (D.Lgs. 26/2014) and the European Legislation (European Directive 2010/63/EU).

Culture preparation was performed as previously described^61^. Briefly, neuronal cultures were obtained from cerebral cortices of embryonic rats, at gestational day 18 (E18). The cerebral cortices of 4–5 rat embryos were dissected and then exposed to chemical (0.125% trypsin solution for 20 minutes at 37 °C) as well as mechanical dissociation (through flame-narrowed Pasteur pipettes). The resulting tissue was re-suspended in Neurobasal medium (Invitrogen, Carlsbad, CA, USA), supplemented with 2% B27^62, 63^ and 1% Glutamax-I (both Invitrogen) at the final concentration of about 1,200 cells/μl. Cells were then plated onto the substrates, pre-coated with adhesion promoting molecules (first laminin 50 μl/ml, and second poly-D-lysine 100 μl/ml, both from Sigma-Aldrich, St. Louis, MO, USA), at the estimated density of 48,000–50,000 cells/device (around 2,000 cells/mm^2^) (see Fig. 1a). The cultures were maintained onto micro-electrode array (MEA, Fig. 1c) devices, each containing 1 ml of nutrient medium (i.e. serum-free Neurobasal medium supplemented with 2% B27 and 1% Glutamax), in a humidified incubator having a controlled atmosphere (5% CO_2_, balance air) at 37 °C. No antimitotic drug was added in our cultures to prevent glia proliferation^64^. Half of the medium was replaced once a week until the 4^th^ week *in vitro* and twice a week afterwards. The cultures could be kept in healthy conditions for several weeks and after 3-4 weeks *in vitro* they reached a mature developmental stage, characterized by quasi-synchronous array-wide bursts, mixed with isolated random spikes (cf. Fig. 1d)^15, 65^.

### Micro-Electrode Array (MEA) experimental setup

Primary cultures of cortical neurons were plated onto arrays (60MEA200/30-Ti, Multi Channel Systems-MCS, Reutlingen, Germany) of 60 planar TiN/SiN micro-electrodes (30 µm diameter, 200 µm spaced, 8 × 8 geometrical layout, see Fig. 1b). The experimental setup was based on the MEA60 system by MCS, consisting of a MEA mounting support with integrated 60 channels pre- and filter amplifier (MEA 1060-Up, gain 1200x) and a personal computer equipped with a PCI data acquisition board for real-time signal monitoring and recording. A temperature controller (TC02, MCS) was used to keep constant the temperature of the sample during the experiment at 37 °C. A custom recording chamber, consisting of a metallic box heated from the topside through planar high-power ceramic resistors (BI Technologies, Fullerton, CA, USA) and providing an inlet for a constant gas flow (5% CO_2_–20% O_2_–75% N_2_), was used to keep proper environmental conditions (temperature, CO_2_ concentration) during the experiment^66^. Moreover, custom-made polydimethylsiloxane (Sylgard 184, Sigma-Aldrich) lids for MEAs were used to reduce water evaporation and maintain the medium’s osmolarity constant during the experiment^67, 68^. Electrophysiological signals were acquired at a sampling rate of 10 kHz through the commercial software MC_Rack (MCS), used also for on-line visualization and raw data storage. Data was further processed by using custom software tools developed in MATLAB (Mathworks Inc., Natick, MA, USA), as described in the next sections. Trains of electrical stimuli were programmed through MC_Stimulus software (MCS) and delivered to different channels of the MEA by STG 4008 stimulator (MCS).

### Dataset

Our dataset comprises recordings from 13 different cultures in basal conditions (i.e. kept in culture medium), coming from 2 cell preparations. All cultures were recorded during the 4^th^ and the 5^th^ week *in vitro*, between 21 and 35 days *in vitro* (DIVs).

### Spike and stimulation artifact detection

The collected data was analyzed off-line by using custom analysis tools developed in MATLAB. High-pass filtered (cut-off frequency 100 Hz, 2^nd^ order Butterworth filter) extracellular recordings of neuronal network activity provide a noisy measurement of the action potentials produced by few neurons (1–3) coupled to each recording electrode (i.e. multi-unit activity). Typical signal amplitudes are in the range of 20–200 μV and are embedded in biological and thermal noise ranging from 5 μV up to 15 μV peak-to-peak. We first detected spikes by using the “precise timing spike detection” method, described in a previous study^69^. The detection and suppression of stimulation artifacts were performed by means of an off-line version of the SALPA (Subtraction of Artifacts by Local Polynomial Approximation) algorithm proposed by Wagenaar and Potter^70^, re-implemented in MATLAB.

### Burst and network burst detection

A ‘burst’ consists of a fast sequence of spikes recorded on a single channel, with a duration equal to the sum of the inter-spike intervals (ISI) within the burst itself and separated by a relatively long interval compared to the burst duration^71^. When the bursting behavior is organized in array-wide barrages involving the entire network at the same time, the phenomenon is usually indicated in the literature by the name of ‘network burst’ (NB)^14^. The burst detection algorithm we used is based on the computation of the logarithmic ISI histogram and automatically detects the best threshold to distinguish between inter- and intra-burst inter-spike intervals channel-by-channel^72^. According to a recent report^73^ this algorithm shows high performances in detecting bursting activity in *in vitro* cultures. An analogous procedure is followed for the detection of NBs, looking for sequences of quasi-synchronous single-channel bursts in at least 20% of active channels (see^72^ for further details). For each spontaneous NB, we saved the rank order of activation of electrodes and time delays with respect to the first firing electrode of the sequence (i.e. leader).

### Major Leader identification

For each NB detected in the spontaneous activity phase we identified the recording channels which NBs mostly originate from, called Major Leaders (MLs), following the procedure described in the literature^30, 31^. Then we derived the histogram of the burst leadership score (LS), by counting for each electrode how many times it was leader of a NB. A long enough period of time (usually few hours, depending on the frequency of NBs) is necessary to get a clear picture of the burst leaders in a culture. Following the work of Ham^30^, we defined MLs as those electrodes leading at least 4% of all NBs. As shown in the Results section, those channels detected as leaders also correspond to the most active.

### Experimental protocol

Spontaneous activity from cultures in the 4^th^–5^th^ weeks *in vitro* was recorded for at least 2 hours before electrical stimulation, after a period of rest in the experimental setup to allow for stabilization outside the incubator (~1 hour). This gave us the possibility to record between 500 and 5000 NBs per culture, depending on the bursting rate. Raw voltage traces were saved and promptly processed in MATLAB for detecting spikes, bursts, NBs and ML channels. Based on this preliminary analysis, we selected the stimulating electrodes: we delivered to each culture a test stimulus from eight channels of the array, four of which were classified as MLs and the rest as non-MLs (i.e., followers). The MLs selected for stimulation were those with the highest LS, whereas the others were selected randomly among active followers (i.e. provided they recorded some activity). A “test stimulus” consisted of a train of monopolar 100 voltage pulses at 0.2 Hz, each of which being a positive-then-negative square wave (amplitude ± 750 mV, duration 500 μs, duty cycle 50%)^74^. Since we wanted to identify unambiguously the stimulation site (being either a ML or a follower), we used monopolar stimulation (i.e. referenced to ground) instead of bipolar stimulation (i.e. referenced to a neighbor micro-electrode). Bipolar stimulation was not ideal in this case, because the choice of the neighbor electrode to use as reference of the stimulation was not univocal. Moreover, we knew from our previous work that also in this configuration network responses are site-dependent, suggesting that the gross effect of stimulation can still be considered local also when referenced to ground^75, 76^. The stimulation frequency had been set at 0.2 Hz based on previous work and literature^75, 77–79^, with the aim of eliciting independent responses in time. When using higher frequencies (i.e. >0.3 Hz), evoked network responses tend to be time-correlated^80^.

Since spontaneous activity is not inhibited by any pharmacological intervention while stimulating, the size and delay of evoked network responses might depend on the ongoing dynamics in a non-trivial way. This could be avoided by using a closed-loop stimulation protocol in which the onset of the stimulation depends on the time past the last spontaneous burst as shown in^81^. However, Shahaf and colleagues^38^ already demonstrated that the activation pattern elicited by different stimulated channels is a conserved feature of the response, also when using an open-loop stimulation protocol. Moreover, our analysis takes into account the fact that a variable percentage of stimuli do not elicit any response or incomplete responses by discarding them or normalizing over the number of activated electrodes.

### Post-stimulus time histogram (PSTH) analysis

The simplest method to characterize the average network response evoked by electrical stimulation is to compute the post-stimulus time histogram (PSTH), which represents the average spike count of each recording site as a function of binned time since stimulus (time window: 500 ms; time bin: 2 ms)^82^. Electrodes showing a PSTH area lower than 1 (i.e. less than 1 spike in 500 ms since stimulus on average) were excluded from further analysis. We considered the PSTH area as a measure of the size of the response for each site of the MEA. Since we wanted to check whether the network response to electrical stimuli delivered from MLs is significantly different than that delivered from followers, we normalized the PSTH area of each responding channel in response to different stimulating sites to the maximum obtained. Then, we compared the statistical distributions of all responses to stimulation from ML and from follower sites. We applied the same procedure also to the delay of the first spike of each response (first-spike latency). We also asked whether MLs respond differently than other channels to electrical stimulation, therefore we performed a second normalization of the PSTH areas. For each stimulating site, we divided each PSTH area by the maximum one among all responding channels. Similarly, we compared the distributions of all responses of ML sites and of follower sites. As before, we applied the same procedure to the first-spike latency.

The spiking responses evoked by local electrical stimulation in dissociated cortical cultures is typically formed by an *early* and fast component (approx. time range 0–35 ms after stimulus), usually involving few channels of the MEA, followed by a *late* and slower component (approx. 35–500 ms), which usually involves the whole array^38, 83^ (cf. Fig. 1e). These two components often overlap in time and their separation is not straightforward. Hence, we devised a method to optimally separate early from late components for each stimulated channel. First, we smoothed the network PSTH (obtained by summing all responding channels’ responses) via moving average (20-ms time bin), and second we detected all peaks (i.e. local maxima). If a peak was found within the first 50 ms (*x*_*peak1*_, corresponding to the *early* phase), and another one between 50 and 500 ms (*x*_*peak2*_, corresponding to the *late* phase), the algorithm then looks for all local minima between them and selects the lowest one (*x*_*min*_). Then, the separation *s*_*j*_ between the two peaks is computed as

1 $${s}_{j}=1-\,\frac{PST{H}_{j}({x}_{min})}{\sqrt{PST{H}_{j}({x}_{peak1})\cdot PST{H}_{j}({x}_{peak2})}}$$

where *PSTH*_*j*_ (*x*) is the smoothed network PSTH function for the *j*-th stimulating electrode. The value of this parameter ideally ranges from 0 (when the peaks are NOT separated) to 1 (when the peaks are perfectly separated). If *s*_j_ overcomes a low pre-defined threshold (empirically estimated, here set at 0.3), *x*_*min*_ is considered as the optimal time threshold to separate early and late response components for the stimulating electrode *j* and it is applied to all responding channels. If either peak cannot be detected or peaks are not sufficiently separated, the corresponding stimulating electrode is not considered for further analysis. The statistical analysis of PSTH areas and first-spike latencies was carried out by considering separately either early or late responses.

We applied the same burst and NB detection algorithms used to analyze the spontaneous activity period to the evoked activity, only by considering the late response component and by using the time thresholds obtained at the previous step to separate it from the early one. Finally, for each evoked NB we saved the activation order of involved electrodes and the corresponding time delays with respect to stimulation.

All the results obtained from the entire dataset were pooled together.

### Pattern distance

For each pair of recorded NBs (either spontaneous or evoked), we computed a similarity index, based on the string *edit distance* ^84, 85^ between the electrode activation orders of the two NBs. In fact, each spatial pattern of burst activity propagation can be assimilated to an ordered string of symbols/characters, each of which associated to a specific recording electrode. To measure the similarity between each pairs of patterns, the *Levenshtein edit distance* ^84, 85^ between the two corresponding strings can be computed as the minimum number of editing operations (insertions, deletions and substitutions) needed to transform one string into the other^38^ (cf. Supplementary Figure 5). This measure depends on the length of the two strings (and it is upper limited by the size of the longest string, if all edit operations have weight equal to 1). Hence, we needed to *normalize* it to be able to compare distances between string pairs of different lengths (cf. Supplementary Figure 5).

To this end, we made use of a surrogate data generation approach. We randomly shuffled each pair of strings (i.e. activity patterns) 200 times and we computed the corresponding edit distances: these values were used to estimate the statistical distribution of chance distances between two strings of the same length as the original ones (i.e. null distribution). Then, a p-value was computed as the ratio of shuffled strings’ distances lower than the original strings’ distance. The lower is the obtained p-value, the higher is the similarity between the two strings. Moreover, p-values can be thresholded according to the desired level of significance (e.g. 0.05), to determine whether patterns are significantly more similar than expected by chance or not (cf. Supplementary Figure 5). This analysis was done separately on both spontaneous and evoked patterns, and between spontaneous and evoked patterns (cf. Supplementary Figure 6). We considered up to a maximum of 2500 spontaneous NBs per experiment. Regarding evoked activity, we selected only those stimulated channels evoking a NB in at least 50% of trials. An evoked NB was detected when stimulation elicited a single-channel burst in at least 20% of active channels (similarly to what was done for detecting spontaneous events). Only responses generated within the first 500 ms since stimulation were considered.

### Pattern clustering

We applied an unsupervised iterative clustering procedure to determine whether either spontaneous or evoked NB patterns could be clustered together according to their similarity. Basically, the number of existing clusters with different activation patterns was first determined following the method proposed by Raichman and Ben-Jacob in 2008^17^ with slight modifications (cf. Supplementary Material). Once the number of different clusters was identified, they were used as *templates*: NBs not included in any cluster were associated via template matching to the cluster they are closer to. All unclassified NBs were discarded (cf. Supplementary Methods and Supplementary Figure 6).

### Cluster evaluation

The ratio of significantly similar patterns’ pairs (i.e. showing distance <0.05) over the total number of pairs has been usually regarded as an index of the global similarity of patterns belonging to the same or coming from different clusters.

### Statistical analysis

Whenever the normality assumption failed (checked by applying Kolmogorov-Smirnov test, p-level = 0.01), nonparametric statistical tests were applied (e.g. Mann-Whitney test, Kruskal-Wallis ANOVA, two-sample Kolmogorov-Smirnov). Exact p-values were reported either in the text or in the figure captions. Unless differently specified, all data are reported as mean ± standard error of the mean. In box plots, the median value and 25^th^–75^th^ percentiles are indicated by the box, mean value is indicated by the small square, whereas whiskers indicate 5^th^–95^th^ percentiles. All statistical analyses have been performed by using either OriginPro v 8.6 (OriginLab Corporation, Northampton, MA 01060, USA) or SigmaPlot v 13 (Systat Software, Inc, San Jose, CA 95131, USA).

### Data Availability

The code and datasets generated during and/or analysed during the current study are available from the corresponding author on reasonable request.

## Electronic supplementary material

Supplementary material
