# Supplementary material for: Stimulation triggers endogenous activity patterns in cultured cortical networks
Source: Sci Rep. 2017 Aug 22;7:9080. doi: 10.1038/s41598-017-08369-0 (PMC5567348; doi:10.1038/s41598-017-08369-0)
Supplement: Supplementary file 1 — Supplementary material [file 41598_2017_8369_MOESM1_ESM.pdf]

Supplementary material

Pasquale V., Martinoia S., Chiappalone M. (2017)

Stimulation triggers endogenous activity patterns in cultured cortical networks

Supplementary Results

Spike sorting results

We asked whether there was a specific tendency for channels classified as major leaders (MLs) to record more than one unit with respect to followers. Therefore, we applied to a sub-set of experiments (6 out of 20) a conventional spike sorting algorithm (cf. Supplementary Methods). The global percentage of channels recording more than one putative neuron is 16.61% (3 neurons 0.96%, 2 neurons 15.65%), of which 11.54% were classified as MLs and the rest as followers. This is very close to the global percentage of MLs (11.64%, cf. following paragraph), meaning that there is no specific tendency for channels classified as MLs to record more than one unit. Moreover, if we only consider the 5 most active channels of each experiment (i.e. 30 channels in total for 6 experiments), only 20% (6 out of 30) record more than a single neuron, of which only 1 is also classified as ML (3.33% over the total). Therefore, we can conclude that no significant statistical difference between the firing activity of ML and follower channels should be due to a tendency for ML to record more than one neuron with respect to followers.

Properties of MLs: spontaneous activity

In our dataset we tested whether the set of MLs in a given culture remains stable across hours of spontaneous activity. Even if the leadership score (LS) (cf. Methods) of individual channels may fluctuate (Supplementary Figure 1a), in all cultures there is a set of channels which are consistently recruited at the beginning of bursts with higher probability with respect to all others (i.e. showing higher LS). These channels correspond to the MLs. The pool of MLs remains approximately constant during medium-term recordings (e.g. up to 24h).

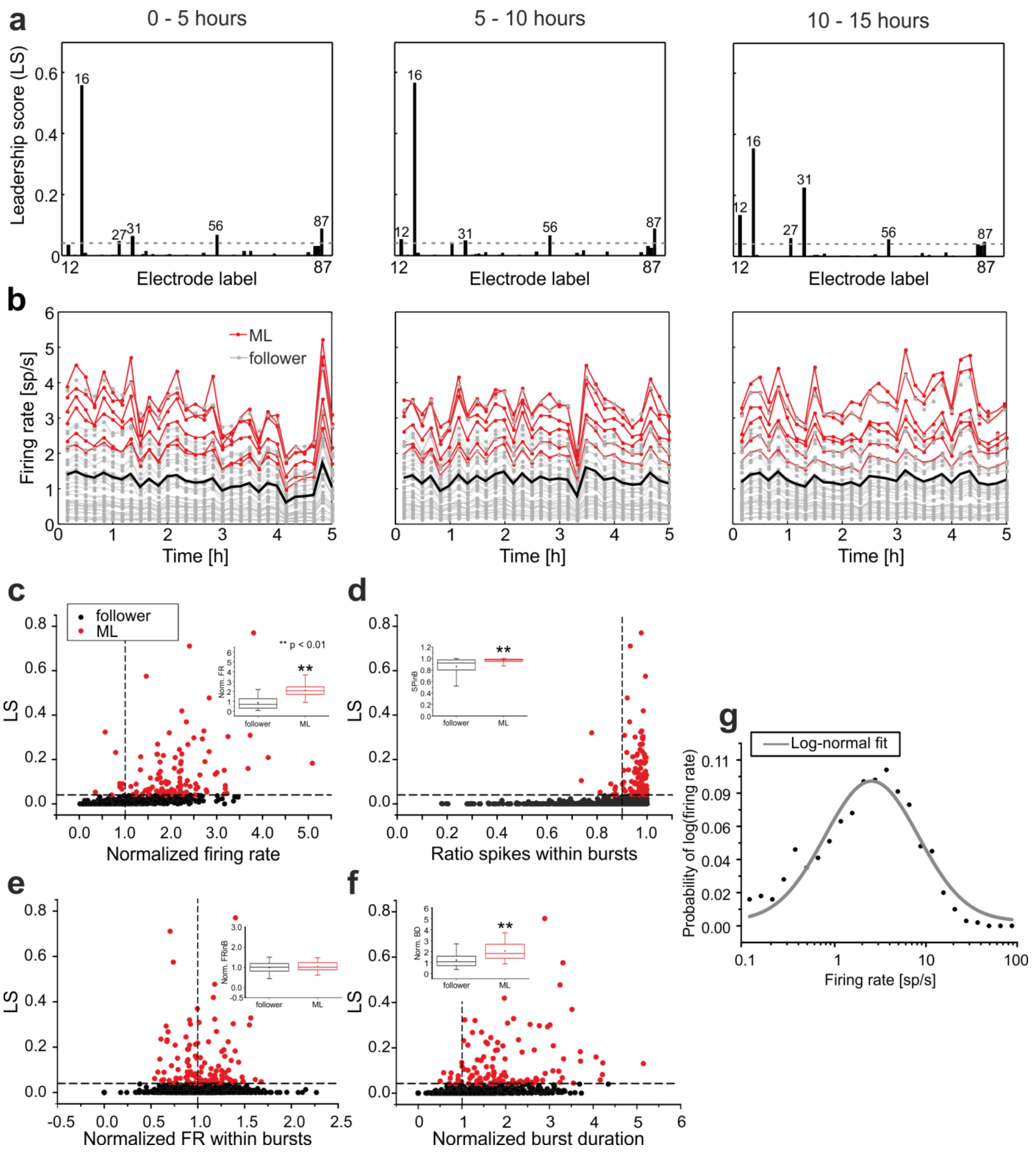

Supplementary Figure 1. Properties of MLs. a, Burst leadership score (LS) of all electrodes during consecutive 5-h time windows (representative experiment). The 4%-threshold is marked by the dotted line and the identified MLs indicated by the electrode labels. **b**, Firing rate (spikes/s) of all recording electrodes during consecutive 5-h time windows for the same representative experiment as in **a** (time bin: 10 minutes): we highlighted in red the MLs. The thick black curve represents the average firing rate over all electrodes. **c**, Burst leadership score (LS) vs. normalized firing rate for the recorded electrodes of the entire dataset. Inset, box plot of statistical distributions of normalized firing rate for both followers (black) and MLs (red), Mann-Whitney U-test $\boldsymbol{p} > |\boldsymbol{U}| = \mathbf{6.41 \cdot 10^{-40}}$. **d**, LS VS ratio of spikes within bursts. Inset, corresponding boxplots for MLs and followers, Mann-Whitney U-test $\boldsymbol{p} > |\boldsymbol{U}| = \mathbf{7.95 \cdot 10^{-13}}$. **e**, LS VS

normalized firing rate within bursts. Inset, corresponding boxplots for MLs and followers, Mann-Whitney U-test $\boldsymbol{p > |U| = 0.38}$. **f**, LS VS normalized burst duration. Inset, corresponding boxplots for MLs and followers, Mann-Whitney U-test $\boldsymbol{p > |U| = 3.20 \cdot 10^{-21}}$. **g**, Probability density of logarithm of spontaneous firing rate (obtained by considering all electrodes of all recordings). In grey, we reported the best log-normal fit, whose equation is $\boldsymbol{f(x) = 2.85 + \frac{1276.10}{\sqrt{2\pi} \cdot 1.18 \cdot x} exp\left(\frac{-ln\left(\frac{x}{10.26}\right)^2}{2 \cdot 1.18^2}\right)}$.

We then analyzed the firing properties of the identified MLs. In Supplementary Figure 1b we reported the firing rate profile of each recording channel of a selected culture during 15-h recording (same culture of Supplementary Figure 1a). We depicted in red the MLs and in grey the followers. The thick black curve represents the network average firing rate. All MLs lie above the network average during the whole recording, but there are also many channels with higher firing rates which are not classified as global MLs. Therefore, we can conclude that MLs, as we defined them, are not only apparent leaders of the bursting activity, but are also highly active.

We also measured other statistics of firing and bursting activity of MLs, namely normalized firing rate, ratio of spikes within bursts, normalized firing rate within bursts, and normalized burst duration (cf. Supplementary Figure 1). We found that MLs feature higher global firing rates, and their activity is almost fully included within bursts, showing longer burst durations, but not higher rates within bursts (cf. Supplementary Figure 1c-f). Those results indicate that MLs are highly active and bursting neurons, and do not show tonic or persistent random spiking activity.

Additionally, inspired by the fact that also *in vivo* a few neurons actually feature higher firing rates than the majority of all others [1-3], leading to a pseudo-lognormal distributions of logarithm of firing rate [4], we derived the same probability density function of logarithm of spontaneous firing rate from our data (considering the whole dataset) and we obtained a very similar relationship. As already shown by the model hypothesized by Roxin and colleagues [4], there is an excess of neurons firing at low rates and a lack of neurons firing at high rates compared with the best lognormal fit (cf. Supplementary Figure 1g).

Evaluation of early/late response component separation

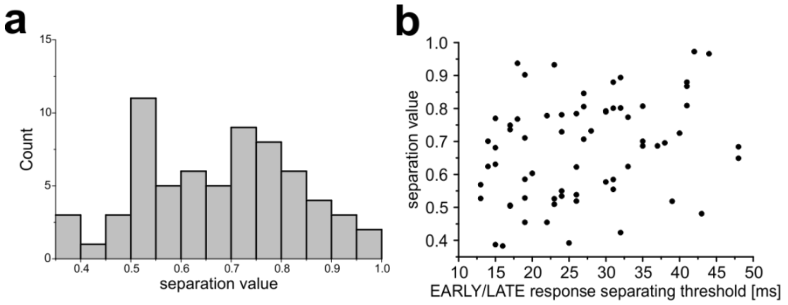

Supplementary Figure 2. Evaluation of early/late response component separation. a, Histogram of separation values (cf. Methods) between early and late response components (bin: 0.5 normalized units). **b**, Scatter plot of separation values vs. early/late response separating time thresholds (Pearson correlation coefficient 0.30, 2-tailed significance test, p-value < 0.05).

In Supplementary Figure 2a we reported the histogram of separation values (cf. Methods) between early and late PSTH response components, which are broadly distributed between 0.35 and 1 (67 stimulating electrodes, 0.68 ± 0.02), and in Supplementary Figure 2b a scatter plot of separation values vs. early/late response separating time thresholds, indicating a low positive correlation (cf. figure legend), meaning that early and late responses tend to be more easily separable when they are farther in time.

Early evoked response of MLs

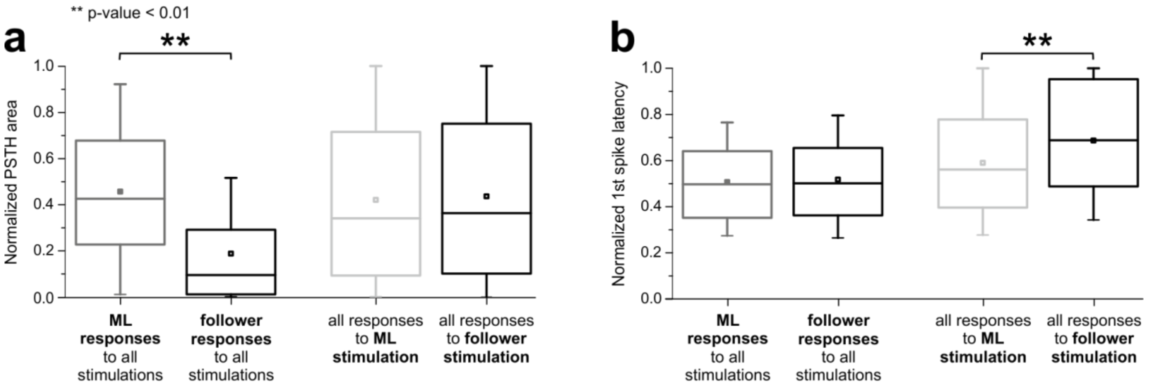

Supplementary Figure 3. Early evoked response of MLs. Box-plots of normalized area (**a**) and normalized first-spike latency (**b**), by considering only the early response. In each panel, on the left: comparison of either ML (grey) or follower (black) responses (area: Mann-Whitney U-test $p > |U| = 0$; latency: Mann-Whitney U-test $p > |U| = 0.43$); on the right: comparison of responses to either ML (light grey) or follower (black) stimulations (area: Mann-Whitney U-test $p > |U| = 0.13$; latency: Mann-Whitney U-test $p > |U| = 3.7 \cdot 10^{-33}$).

As also detailed in the main text, MLs show stronger responses to stimulation than followers in the early phase. This also confirms the fact that MLs tend to be highly active, both in spontaneous and evoked activity periods. Moreover, when stimulation is delivered from MLs, responses show shorter latencies (both early and late, cf. Results section in the main manuscript), indicating that the network is more promptly entrained by ML than by follower stimulation (cf. Supplementary Figure 3).

Spontaneous patterns constrain evoked patterns: multidimensional scaling results

We also used an alternative method to visualize and quantify the similarity between spontaneous and evoked patterns, which does not rely on previous clustering results. Similarly to what had been done in [5] we applied multidimensional scaling (MDS) [6] to the full matrix of all patterns' distances, in order to get a reduced 2D representation of NB patterns, given their multidimensional pairwise distances (cf. Supplementary Methods). In fact, MDS is a nonlinear method used to represent high-dimensional datasets in a low-dimensional (typically 2D) space such that pairwise distances are preserved as well as possible (i.e. points which are close in the original high-dimensional space will also be placed close by in the 2D projection) [5].

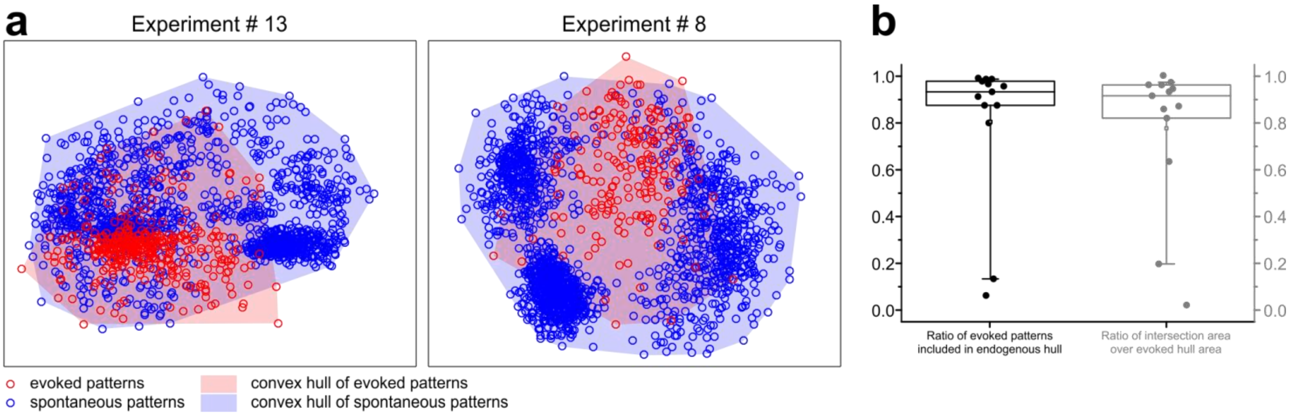

Supplementary Figure 4. MDS analysis. a, Reduced 2D representation of spontaneously generated and evoked bursting patterns (each one represented by either a blue or a red circle, respectively) according to MDS analysis for two selected experiments. Convex hulls are defined as polygons in a 2D space including all considered points. **b**, Box-plot representation of the ratio of evoked patterns included in the hull defined by endogenous ones (black) and of the ratio of the intersection area (between the two hulls) over the evoked hull area (grey).

In Supplementary Figure 4a we reported the results of MDS for two representative experiments. Spontaneous patterns are depicted in blue, while evoked patterns in red. For most experiments, there is a remarkable overlap of the realms including blue and red points. Moreover, we observed a striking qualitative correspondence between the results of our pattern clustering method (cf. Methods) and the results of the MDS analysis, e.g. in the number of separate spontaneous clusters of patterns, although we did not quantify this parameter from MDS.

Taking advantage of MATLAB functions, we first determined the 2D convex hulls including spontaneous and evoked patterns (shaded blue and red areas). Then we measured two quantities, i.e. the ratio of evoked patterns included in the hull defined by endogenous ones and the ratio of the intersection area (between the two hulls) over the evoked hull area. Both measures are reported in Supplementary Figure 4b. Apart from a few cases (i.e. 2 out of 13), the global results highlight that spontaneous and evoked patterns' realms substantially overlap, meaning that endogenous emerging patterns constrain possible evoked patterns.

Supplementary Methods

Spike sorting

The results presented in the main manuscript were obtained by applying no spike sorting procedure.

This choice was made according to many other published studies [7-10], also reporting that in such cultured networks during bursts the global increase of activity, in the form of fast sequences of overlapping spikes, makes the sorting procedure difficult and possibly unreliable.

However, to further confirm the validity of the obtained results, we analyzed a subset of experiments (6 out of 20) by applying a conventional spike sorting algorithm based on wavelet transform and super-paramagnetic clustering, as proposed by Quian Quiroga and colleagues [11]. The MATLAB tool *Wave_clus* (v 2.0) was downloaded from http://www2.le.ac.uk/departments/engineering/research/bioengineering/neuroengineering-lab/spike-sorting and integrated in our software. Briefly, for each detected spike, 32 samples (i.e. 3.2 ms @ 10 KHz)

were saved for further analysis. All spikes were aligned to their minimum at data point 10. In order to avoid spike misalignments due to low sampling, spike minima were determined from interpolated waveforms of 64 samples, using cubic splines. Channels were considered for sorting if recording at least 50 spikes during the whole experiment. First, unit clusters were determined by analyzing the first 30-minute recordings (and considering no more than 20,000 spikes). All other spikes detected during the recording were assigned via template matching, i.e. they were assigned to the closest cluster unless they were too far (threshold: 3 times standard deviation from the cluster center). The obtained results were further refined and confirmed by visual inspection of an expert user. Unclustered spikes were not considered.

Shuffling

Given the non-parametric statistical test based on surrogate data applied to check significance of patterns' pair similarity (cf. *Pattern distance*), the probability of getting two patterns similar by chance should not exceed the chosen significance level (e.g. 0.05). Hence, every time the ratio of significantly similar patterns' pairs exceeds that threshold, it should be considered significant. To further check whether the ratio of significantly similar patterns found in our experiments is statistically significant (i.e. higher than expected by chance), we shuffled spontaneous patterns, by randomly permuting electrode activation rank orders 10 times for each experiment. Then we computed the corresponding global ratio of similar patterns' pairs within shuffled spontaneous activity and between shuffled spontaneous and actual evoked activity (average ± s.d. over the 10 shuffled datasets for each experiment). Due to the computational demand, we limited the number of considered patterns per experiment to 500, and we computed the normalized distance (cf. *Pattern distance*) based on 50 permutations per pair instead of 200. Since this might influence our results, for a subset of experiments (2 out of 13), we kept the number of permutations used to estimate the normalized distance equal to 200, and we did not find significantly different results (always lower than the significance level of 0.05).

Normalization of pattern distance

Normalized distances following the approach described in the main manuscript (and illustrated in Supplementary Figure 5a) do not depend on pattern length and, when re-ordered according to the pattern leader, nicely cluster along the diagonal. In Supplementary Figure 5b we reported a representative example of the effect of normalization on pattern distance values. Upper panels show raw distance matrices, whereas lower panels depict normalized distances, re-ordered either according to pattern length (left) or to pattern leader (right). It is evident that raw distances strongly depend on pattern length, preventing clusters of similar patterns to be detected, whereas normalized distances do not.

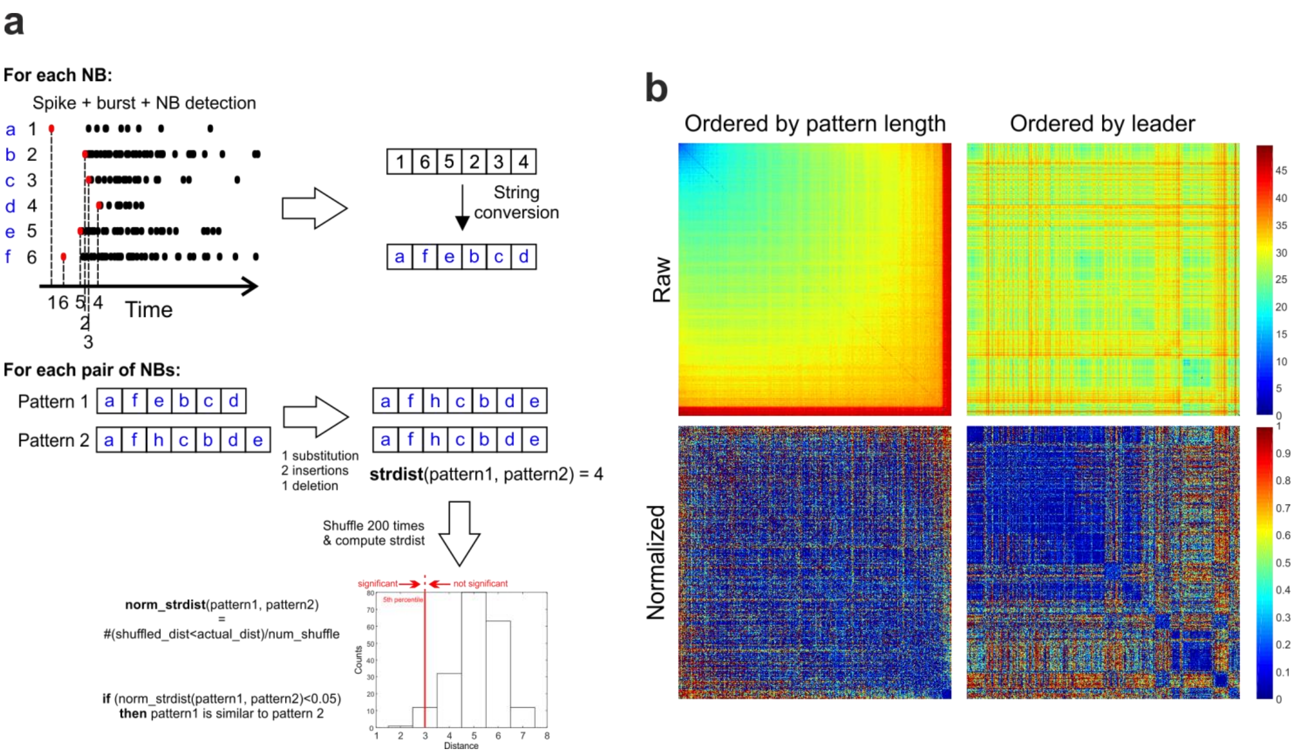

Supplementary Figure 5. Normalization of pattern distance helps identifying clusters regardless of pattern length. a, Scheme illustrating the algorithm for pattern distance computation. Each sequence of electrode first activations during a NB is translated into a string of characters, each of which corresponding to a unique electrode number. Then, for each pair of strings, the Levenshtein edit distance is computed, as the minimum number of editing operations needed to transform one string into the other one. Finally, normalization of the raw string edit distances is made by generating surrogate data via shuffling of the original strings, and counting the number of times the distance between shuffled strings (shuffled_dist.) is less than the distance between original strings (actual_dist) over the total number of shuffles (num_shuffle, e.g. 200). Such normalized distance is equivalent to the p-value of a non-parametric statistical test based on surrogate data, which can be thresholded according to the desired significance level (e.g. 0.05). **b**, Color-coded representation of raw (top) and normalized (bottom) distance matrices among all pairs of spontaneous NBs (representative experiment), either ranked by their pattern length (left) or by their leader (right). Raw distances strongly depend on pattern length, whereas normalized distances are mostly independent on the string length.

Additional information on pattern clustering

According to [12], the distance matrix is first rearranged by a standard agglomerative dendrogram method, based on Euclidean distance (provided by the Statistics Toolbox in Matlab). Second, the algorithm iteratively looks for sets of consecutive *N* patterns in the re-ordered matrix that satisfy two conditions: *separation*, i.e. a pattern belonging to a given set will have the lowest average distance with patterns belonging to the same set than to any other set, and *isolation*, i.e. no pattern will belong to more than one set. The parameter *N* is chosen equal to the square root of the total number of NBs included in the analysis, as suggested in the original paper [12]. Hence, the *k* selected sets (i.e. clusters) do not overlap and they maximize the inner similarity (i.e. minimize the inner distances among patterns) (cf. Supplementary Figure 6). Once the number of different clusters is identified, they are used as *templates* and NBs not included in any cluster are associated via template matching to the cluster they are closer to (i.e. a NB is included in a cluster if its average distance from the patterns belonging to that cluster is less than the average plus three times the standard deviation of that cluster's inner distances). The clustering procedure is applied separately to both spontaneous and evoked activity. In case of evoked patterns, the procedure is applied independently to each stimulated channel's responses, and only one cluster per channel is considered for further analysis (the one including the highest number of responses). All unclassified NBs (i.e. that were not included in a cluster after template matching) were discarded. The percentages of selected NBs are 67.8 ± 2.4 % and 63.3 ± 3.4 % for spontaneous and evoked activity, respectively. Then, the normalized edit distance (cf. previous paragraph, *Pattern distance*) can be computed for each pair of selected spontaneous and evoked patterns.

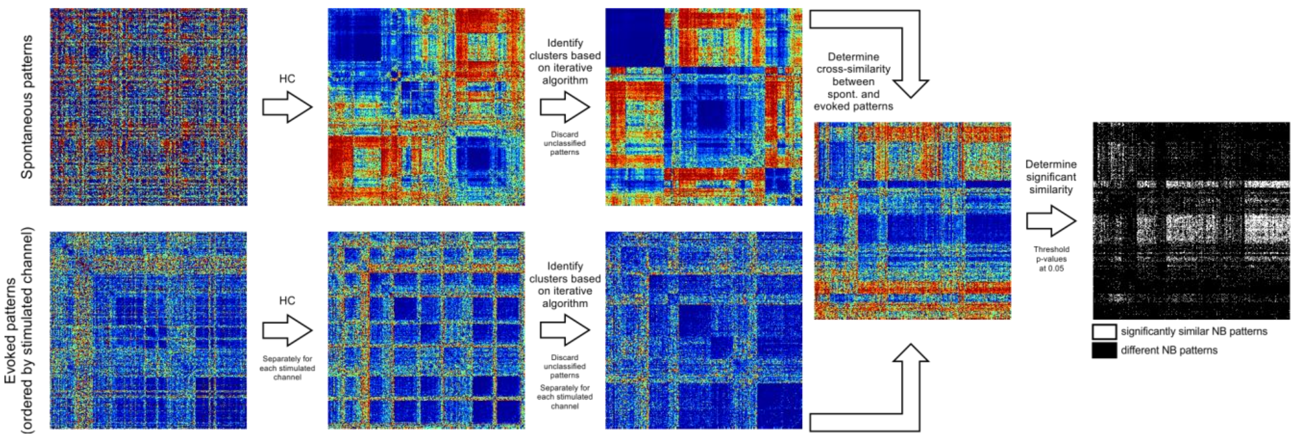

Supplementary Figure 6. Scheme illustrating the clustering algorithm. Both spontaneous and evoked NBs distance matrices are re-arranged according to a standard hierarchical clustering approach, then an iterative algorithm is applied to find clusters of close-by patterns. Patterns that cannot be included into any of the identified clusters according to a user-defined threshold are discarded. That procedure is applied separately to NBs evoked by different stimulation sources. Once spontaneous and evoked patterns are selected, the cross-similarity matrix collecting all distances between spontaneous and evoked NBs is computed. Again, normalized distances can be thresholded to the desired level to determine significant similarity between each pair of patterns.

Multi-dimensional scaling (MDS)

Non-classical metric multidimensional scaling (MDS) was performed in MATLAB [6]. For this analysis we considered up to a maximum of 2500 spontaneous patterns and all evoked patterns, as also done for previous analyses on pattern similarity. MDS was applied to the full matrix of normalized edit distances between all pairs of patterns (spontaneous and evoked). We plotted the results of MDS in two dimensions, and we used the `convhull` function (provided by Matlab, useful to determine the convex hull of a set of points in 2-D space) to find the convex polygons delimiting either spontaneous or evoked patterns. We then used the `inpolygon` function (also provided by Matlab) to determine the ratio of evoked patterns included in the hull defined by spontaneous patterns (separately for each experiment). We then transformed our plot into a binary image (using `poly2mask`, Image Processing Toolbox, Matlab) where pixels included within hulls are non-zero, to compute the intersection area between hulls for each experiment.

References

1 Hromadka, T., Deweese, M. R. & Zador, A. M. Sparse representation of sounds in the unanesthetized auditory cortex. *PLoS biology* **6**, e16, doi:10.1371/journal.pbio.0060016 (2008).

2 Barth, A. L. & Poulet, J. F. Experimental evidence for sparse firing in the neocortex. *Trends in neurosciences* **35**, 345-355, doi:10.1016/j.tins.2012.03.008 (2012).

3 O'Connor, D. H., Peron, S. P., Huber, D. & Svoboda, K. Neural activity in barrel cortex underlying vibrissa-based object localization in mice. *Neuron* **67**, 1048-1061, doi:10.1016/j.neuron.2010.08.026 (2010).

4 Roxin, A., Brunel, N., Hansel, D., Mongillo, G. & van Vreeswijk, C. On the distribution of firing rates in networks of cortical neurons. *The Journal of neuroscience : the official journal of the Society for Neuroscience* **31**, 16217-16226, doi:10.1523/JNEUROSCI.1677-11.2011 (2011).

5 Luczak, A., Bartho, P. & Harris, K. D. Spontaneous events outline the realm of possible sensory responses in neocortical populations. *Neuron* **62**, 413-425, doi:10.1016/j.neuron.2009.03.014 (2009).

6 Kruskal, J. B. & Wish, M. *Multidimensional scaling*. Vol. 11 (Sage, 1978).

7 Chiappalone, M., Massobrio, P. & Martinoia, S. Network plasticity in cortical assemblies. *The European journal of neuroscience* **28**, 221-237, doi:10.1111/j.1460-9568.2008.06259.x (2008).

8 Rolston, J. D., Wagenaar, D. A. & Potter, S. M. Precisely timed spatiotemporal patterns of neural activity in dissociated cortical cultures. *Neuroscience* **148**, 294-303, doi:10.1016/j.neuroscience.2007.05.025 (2007).

9 Shahaf, G. & Marom, S. Learning in networks of cortical neurons. *The Journal of neuroscience : the official journal of the Society for Neuroscience* **21**, 8782-8788 (2001).

10 Wagenaar, D. A., Pine, J. & Potter, S. M. An extremely rich repertoire of bursting patterns during the development of cortical cultures. *BMC neuroscience* **7**, 11, doi:10.1186/1471-2202-7-11 (2006).

11 Quiroga, R. Q., Nadasdy, Z. & Ben-Shaul, Y. Unsupervised spike detection and sorting with wavelets and superparamagnetic clustering. *Neural computation* **16**, 1661-1687, doi:10.1162/089976604774201631 (2004).

12 Raichman, N. & Ben-Jacob, E. Identifying repeating motifs in the activation of synchronized bursts in cultured neuronal networks. *Journal of neuroscience methods* **170**, 96-110, doi:10.1016/j.jneumeth.2007.12.020 (2008).
